# Temperature‐sensitive sodium beta‐glycerophosphate/chitosan hydrogel loaded with all‐trans retinoic acid regulates Pin1 to inhibit the formation of spinal cord injury‐induced rat glial scar

**DOI:** 10.1002/btm2.10729

**Published:** 2024-10-17

**Authors:** Rongmou Zhang, Ting Tang, Huafeng Zhuang, Peiwen Wang, Haiming Yu, Hao Xu, Xuedong Yao

**Affiliations:** ^1^ Department of Orthopaedics The Second Affiliated Hospital of Fujian Medical University Quanzhou China; ^2^ Department of Neurology The Second Affiliated Hospital of Fujian Medical University Quanzhou China

**Keywords:** ATRA, glial scar, PI3K/AKT/CDK2, Pin1, sodium beta‐glycerophosphate/chitosan hydrogel

## Abstract

Glial scar formation is a major obstacle to nerve regeneration following spinal cord injury (SCI). Pin1 and the PI3K/AKT/CDK2 signaling pathway play crucial roles in neuronal regulation, but research on their involvement in glial scarring remains limited. In this study, we have for the first time observed that Pin1, PI3K, AKT, and CDK2 are upregulated and interact with each other following SCI. Further experiments revealed that Pin1 contributes to the development of glial scars by promoting astrocyte proliferation, inhibiting apoptosis, and activating the PI3K/AKT/CDK2 pathway. Additionally, all‐trans retinoic acid (ATRA), a specific chemical inhibitor of Pin1, effectively suppresses Pin1 expression. However, its clinical application is limited by its short half‐life and susceptibility to inactivation. To address these issues, we have developed a thermosensitive sodium beta‐glycerophosphate (β‐GP)/chitosan (CS) hydrogel loaded with ATRA (β‐GP/CS@ATRA). This hydrogel exhibits favorable morphology and biocompatibility. Compared to free ATRA, the β‐GP/CS@ATRA hydrogel significantly enhances functional motor recovery after SCI and protects spinal cord tissue, thereby inhibiting glial scar formation. Mechanistically, ATRA administration blocks the development of glial scars and the activation of the PI3K/AKT/CDK2 pathway by inhibiting Pin1 expression. This study suggests that combining ATRA with a hydrogel to target Pin1 expression may be a promising strategy for treating glial scar formation following SCI.

**Translational Impact Statement**

This research introduces novel findings that Pin1 may serve as a potential therapeutic target for mitigating glial scar formation post‐SCI. Furthermore, we successfully prepared β‐GP/CS@ATRA, an ATRA‐loaded temperature‐sensitive β‐GP/CS hydrogel, for the first time. In vivo investigations demonstrated that β‐GP/CS@ATRA impedes the development of glial scars and the activation of the PI3K/AKT/CDK2 pathway by suppressing Pin1 expression, exhibiting favorable biocompatibility. The β‐GP/CS@ATRA hydrogel exhibits promising applications, representing a prospective targeted treatment for glial scar formation after SCI.

## 1 INTRODUCTION

Spinal cord injury (SCI) represents one of the most severe types of neural injuries^1^ and is the second leading cause of paralysis, following strokes.^2^ SCI primarily results from damage to axons along the spinal cord, leading to the loss of motor, sensory, and autonomic nerve functions below the injury site.^3^ This profoundly impacts the quality of life for individuals with SCI. Global reports indicate an annual occurrence of approximately 250,000–500,000 SCI cases.^4^ Over the past 30 years, the global incidence of SCI has risen from 236 cases per million people to 1298 cases per million.^5^ Following SCI, injury activation occurs in resident spinal cord astrocytes and meningeal cells, recruiting infiltrating fibroblasts and Schwann cells from the peripheral nervous system. This process leads to the formation of neural glial scars within the damaged spinal cord.^4^, ^6^ Glial scars significantly impede axonal regeneration and myelination, thereby influencing neural regeneration post‐SCI.^7^, ^8^, ^9^ Consequently, the formation of glial scars represents a critical therapeutic target for neural regeneration after SCI. However, effective treatments specifically targeting the formation of glial scars post‐SCI are currently lacking.

Peptidyl‐prolyl cis‐trans isomerase NIMA‐interacting 1 (Pin1) was initially discovered in 1996 and belongs to a subtype of peptidyl‐prolyl cis‐trans isomerases (PPIases). Pin1 can bind and catalyze the cis/trans isomerization of phosphorylated serine/threonine‐proline motifs, leading to protein phosphorylation, degradation, as well as neuronal survival and death.^10^ Its regulatory role in phosphorylation and transcription landscapes positions this enzyme as a central effector in numerous physiological and pathological activities associated with neuronal cells.^11^ Current evidence suggests a close association between Pin1 and various neuro‐related diseases such as epilepsy,^12^ Parkinson's,^13^ Alzheimer's,^14^ and Huntington's diseases.^15^ In the context of SCI, Pin1 has been demonstrated to protect the spinal cord by reducing the apoptosis of oligodendrocytes,^16^ indicating its significant role in the development of SCI. However, the impact and underlying mechanisms of Pin1 on the formation of glial scars post‐SCI remain unclear. Moreover, following SCI, the PI3K/AKT pathway is activated. Activation of the PI3K/AKT pathway can prevent oxidative stress, inflammation, and cell death while inhibiting this pathway can prevent the formation of glial scars.^17^, ^18^, ^19^ Previous studies have shown a positive correlation between Pin1 expression and the activation of the PI3K/AKT signaling in SCI.^20^ Pin1 can phosphorylate the Ser434 site of AKT, increasing AKT stability, and thereby activating PI3K/AKT,^21^ subsequently activating CDK2.^22^ Additionally, Pin1 can alleviate the inhibition of CDK2 by the CDK inhibitor p27.^23^ These findings suggest that Pin1 may interact with the PI3K/AKT/CDK2 pathway, thereby participating in the development of glial scars. However, the specific role of Pin1 in conjunction with the PI3K/AKT/CDK2 pathway in the formation of neural glial scars warrants further elucidation.

All‐trans retinoic acid (ATRA) is one of the active derivatives of vitamin A^24^ and, concurrently, a novel specific chemical inhibitor of Pin1.^25^ ATRA selectively inhibits and degrades active Pin1 in cells by mimicking the psER/Thr‐Pro gene sequence present in Pin1 substrates. This structural mimicry induces the carboxyl group within ATRA to form a salt bridge with Pin1 catalytic residues, thereby exerting its inhibitory effects.^25^ In diseases such as cancer, ATRA is commonly employed to suppress Pin1 expression and activity.^26^ However, the limitations of ATRA, including low water solubility, sensitivity to light, heat, and oxidants, as well as a short half‐life (merely 45 min), contribute to suboptimal efficacy in Pin1 inhibition.^27^ To overcome these drawbacks, there is a necessity to develop new formulations capable of delivering ATRA to its target at a sustained rate while preserving its activity and stability.^24^

Currently, to overcome the limitations of ATRA and effectively deliver it to target tissues, various nanoformulations have been successfully developed, including nanoliposomes, polymer nanocomposites, and ATRA nanoparticles. While these nanoformulations enhance the therapeutic effects of ATRA, their preparation processes are time‐consuming and often involve additional steps to remove organic solvents, impacting the activity of ATRA.^28^ In comparison to nanoformulations, hydrogels have garnered widespread attention due to their excellent biocompatibility, ease of preparation, low cost, and low toxicity.^29^ Meanwhile, hydrogels possess a highly porous structure, facilitating drug release.^30^ In biomedical applications, the most commonly used hydrogels are primarily composed of chitosan (CS) and sodium beta‐glycerophosphate (β‐GP). Both components exhibit good biocompatibility and can form thermosensitive hydrogels at body temperature.^29^ CS is a unique cationic polysaccharide soluble in weak acidic solutions. When sodium β‐glycerophosphate (β‐GP) is added to an acidic CS solution, a temperature‐sensitive hydrogel (β‐GP/CS hydrogel) is formed.^31^ The thermosensitive properties of β‐GP/CS hydrogel lead to gelation at physiological temperatures (36–37°C), making it an ideal drug delivery system.^32^ Additionally, β‐GP/CS hydrogel exhibits suitable mechanical properties for sustained delivery of therapeutic molecules.^33^

In this study, we investigated the in vitro and in vivo effects of Pin1 on the formation of glial scars. Additionally, we encapsulated ATRA for the first time into β‐GP/CS hydrogels and further assessed the in vivo impact of Pin1 inhibition on glial scar formation after SCI. The research findings indicate that Pin1 promotes the formation of glial scars after SCI and activates the PI3K/AKT/CDK2 pathway. Simultaneously, the CS/β‐GP hydrogel loaded with ATRA, by reducing Pin1 levels in vivo, inhibits the formation of glial scars and the activation of the PI3K/AKT/CDK2 pathway, demonstrating excellent biocompatibility. These results suggest that Pin1 may serve as a potential target for treating glial scar formation after SCI, and the β‐GP/CS@ATRA hydrogel could be a promising targeted therapeutic agent for addressing glial scar formation post‐SCI.

## 2 MATERIALS AND METHODS

### 2.1 Experimental animals and ethics

A total of 144 healthy male Sprague–Dawley (SD) rats, aged 8–10 weeks and weighing 260–320 g, were purchased from the Animal Center of Fujian Medical University. All animal experimental procedures in this study complied with the Guide for the Care and Use of Laboratory Animals. The experimental protocol received approval from the Animal Experiment Ethics Committee of the Second Affiliated Hospital of Fujian Medical University (Approval No. [2020] Fuzhou Second Hospital Ethics Review No. 91).

### 2.2 In vivo and in vitro expression and interaction of Pin1 and the PI3K/AKT/CDK2 pathway in SCI

#### 2.2.1 Establishment of SCI rat model

A total of 48 SD rats were randomly divided into the control group (Control) and the model group (SCI), with 24 rats in each group. Rats in the SCI group were anesthetized by intraperitoneal injection of 3% sodium pentobarbital (30 mg/kg). Subsequently, a laminectomy was performed at the T9–T10 vertebral level to expose the spinal cord. Microvascular clamps were then used to compress the spinal cord for 90 s (20 g/cm^2^). After surgery, the incision was sutured layer by layer, and the wound was disinfected with 70% ethanol. Rats in the Control group underwent laminectomy only. Postoperatively, all rats were placed in warm cages for recovery and provided with food and water. Additionally, on postoperative days 1, 3, 10, and 21, three rats from each group were randomly selected and euthanized, and the T9‐T10 spinal cord segment was collected, and frozen at −80°C for subsequent experiments.

#### 2.2.2 Quantitative real‐time polymerase chain reaction (qRT‐PCR)

Total RNA was extracted from tissues or cells using Trizol reagent. The reverse transcription kit and real‐time fluorescence quantitative PCR kit instructions were followed for subsequent procedures, with GAPDH used as an internal reference. The relative expression levels of Pin1, PI3K, AKT, and CDK2 were calculated using the 2^–ΔΔCT^ method by the manufacturer's protocols. The primer sequences are listed in Table 1.

**TABLE 1:** The primer sequence.

Gene name	Forward	Reverse
Pin1	5′‐GTGCCTTCAGCAGAGGTCAGA‐3′	5′‐GCGTCCATGGGCTGTATGTG‐3′
PI3K	5′‐ACAAGAGTTTCCTGGGCATCAA‐3′	5′‐CCTAACGCAGACATCCTGGAA‐3′
AKT	5′‐CCGCCTGATCAAGATGACAGC‐3′	5′‐CCCAGCACATCCGAGAAACA‐3′
CDK2	5′‐ACGGACGGAGCTTGTTATCTCAAAT‐3′	5′‐CAGGTGAAGAGGGCTTTGGG‐3′
GAPDH	5′‐GGCTACACTGAGGACCAGGT‐3′	5′‐GCTGTAGCCATATTCATTGT‐3′

#### 2.2.3 Western blot assay

Total protein was extracted from tissues or cells using RIPA lysis buffer, and the concentration of total protein was determined using the BCA assay kit. Protein samples were separated on a 10% sodium dodecyl sulfate‐polyacrylamide gel electrophoresis gel and subsequently transferred to a polyvinylidene difluoride (PVDF) membrane. The PVDF membrane was then incubated overnight at 4°C with Pin1 antibody (1:2000), PI3K antibody (1:200), AKT antibody (1:5000), CDK2 antibody (1:1000), Bax antibody (1:2000), Bcl2 antibody (1:2000), PCNA antibody (1:5000), p‐PIK3 antibody (1:200), p‐AKT antibody (1:500), p‐CDK2 antibody (1:1000), β‐actin (1:1000) and GAPDH (1:5000). After incubation with the respective secondary antibodies at room temperature for 1 h, protein bands were visualized using the ultra‐sensitive ECL chemiluminescence kit, and quantitative analysis was performed using Image J software.

#### 2.2.4 Co‐immunoprecipitation (Co‐IP)

Co‐IP was employed to assess the interactions between Pin1 and PI3K, AKT, and CDK2. Tissue samples were placed in lysis buffer and homogenized using a homogenizer. After centrifugation, the supernatant was collected and incubated with Pin1 antibody, PI3K antibody, AKT antibody, CDK2 antibody, and IgG antibody. Protein A/G beads were then added, and Western blot analysis was performed to detect the interactions between the proteins.

#### 2.2.5 Immunofluorescence

Paraffin‐embedded spinal cord tissue sections or cells were incubated overnight at 4°C with a primary antibody against Pin1 (Abcam). Subsequently, the sections were incubated with a corresponding secondary antibody at room temperature for 1 h in the dark. Next, the sections were counterstained with DAPI for 10 min. Finally, images were captured using a confocal microscope.

#### 2.2.6 Primary astrocyte culture

As mentioned earlier,^34^, ^35^ primary astrocytes were isolated from the spinal cord tissue of SD rats. In brief, SD rats were euthanized, and their lumbar spinal cord tissues were dissected, dissociated, minced, and digested. The digestion was terminated by adding complete culture medium containing 10% fetal bovine serum (FBS). After centrifugation to collect the cell pellet, it was resuspended to prepare a cell suspension. The cell suspension was seeded into culture flasks containing culture medium with 10% FBS and incubated at 37°C in a 5% CO_2_ incubator until cells reached confluence, with medium changes every 2–3 days. Non‐astrocytic cells were removed by overnight shaking at 37°C and 200 rpm, followed by removal of the medium and replacement with fresh culture medium. Isolated astrocytes were seeded into new culture flasks, and upon reaching a confluence of over 90%, they were passaged. Mature astrocytes were obtained after three passages and collected for subsequent experiments.

#### 2.2.7 Adenovirus transfection

Recombinant adenoviruses carrying Pin1 overexpression (Ad‐Pin1) were designed by Genechem, with adenoviruses carrying an empty gene serving as a control (Ad‐Ctrl). Astrocytes were seeded at a concentration of 4 × 10^4^ cells/mL in a 6‐well plate containing 2 mL of culture medium. The plate was then placed in an incubator for cultivation. When the cell confluence reached approximately 40%–50%, the cells were removed. The corresponding adenovirus was added along with 1 mL of culture medium and incubated for 12 h in the incubator. Finally, the transfection efficiency was assessed using qRT‐PCR and Western blot assay.

#### 2.2.8 Enzyme‐linked immunosorbent assay (ELISA)

According to the manufacturer's instructions, the ELISA Kit (Solarbio Science & Technology Co., Ltd., Beijing, China) was used to detect the expression levels of Collagen I and Collagen III in astrocyte or blood samples.

### 2.3 Synthesis and characterization of β‐GP/CS@ATRA hydrogel

#### 2.3.1 Preparation of β‐GP/CS@ATRA hydrogel

Weigh 2 g of CS powder and dissolve it in 100 mL of 0.1 M acetic acid solution. Stir the mixture at room temperature for 4 h under magnetic stirring to obtain a chitosan solution with a mass fraction of 2% (w/v). Subsequently, weigh 10 mg of ATRA and dissolve it thoroughly in the CS solution. Take 18.0 g of β‐GP powder, add it to 30 mL of distilled water, and sonicate for 10 min to dissolve. Filter the solution through a 0.22 μm filter to obtain a β‐GP solution with a mass fraction of 60% (w/v). Simultaneously, cool the CS@ATRA solution and the β‐GP solution in an ice‐water bath for half an hour. Then, transfer 70 mL of the CS@ATRA solution to a glass bottle and slowly add 30 mL of the β‐GP solution. Stir the mixture under magnetic stirring in an ice‐water bath for 15 min. Finally, the β‐GP/CS@ATRA solution can be obtained.

#### 2.3.2 Characterization of β‐GP/CS@ATRA hydrogel

The β‐GP/CS and β‐GP/CS@ATRA samples were rapidly frozen in liquid nitrogen and subsequently sectioned to obtain cross‐sections. The obtained cross‐sections were then subjected to freeze‐drying. The resulting dried cross‐sections were affixed onto a conductive adhesive and examined for morphological characteristics using a scanning electron microscope (SEM) (SU8010, Japan). Additionally, the rheological performance of β‐GP/CS@ATRA was investigated using a rotational rheometer (DHR‐2, USA).^36^

#### 2.3.3 In vitro drug release

The drug release of β‐GP/CS@ATRA was determined using the dialysis bag method. A total of 10 mL of β‐GP/CS@ATRA was placed inside a dialysis bag and immersed in 100 mL of PBS (pH = 7.4). The dialysis bags were then subjected to simulated release conditions by placing them on an oscillating platform at both 4°C and 37°C. At specific time intervals (0, 1, 2, 4, 6, 8, 12, 18, 24, 36, and 48 h), 10 μL of the released liquid was withdrawn. The concentration of ATRA was determined using high‐performance liquid chromatography (HPLC) (Agilent 1260, USA), and release curves were plotted.

### 2.4 Inhibition of glial scar formation in SCI rats by in vivo application of β‐GP/CS@ATRA

#### 2.4.1 Subcutaneous transplantation of β‐GP/CS@ATRA

Following the previously described methods, we established SCI rat models. After successful modeling, 96 SCI rats were randomly divided into SCI group (no treatment), ATRA group (treated with ATRA), β‐GP/CS group (treated with β‐GP/CS), and β‐GP/CS@ATRA group (treated with β‐GP/CS@ATRA), with 24 rats in each group. The rats in the SCI group, ATRA group, β‐GP/CS group, and β‐GP/CS@ATRA group were subcutaneously injected with 100 μL of PBS, ATRA, β‐GP/CS, and β‐GP/CS@ATRA, respectively, where the drug concentration of ATRA and β‐GP/CS@ATRA was 5 mg/kg. On days 1, 3, 10, and 21 after treatment, three rats were randomly selected from each group for euthanasia. The spinal cord tissues of the rats' T9–T10 segments were collected and stored at −80°C for subsequent experiments. ELISA tests and western blot assay were performed on selected spinal cord tissue samples from each group to assess the expression levels of Collagen I, Collagen III, Pin1, PI3K, AKT, and CDK2 in the spinal cord tissue. The experimental procedures for ELISA tests and western blot assay followed the aforementioned steps.

#### 2.4.2 The assessment of motor function recovery

Rats were placed in an open field (120 cm × 200 cm), and hindlimb movements were observed. Motor function recovery of the hindlimbs in SCI rats was assessed on postoperative days 1, 3, 10, and 21 using the Basso‐Beattie‐Bresnahan (BBB) locomotor rating scale, with a scoring range from 0 (no movement) to 21 (normal gait). The assessment was performed by three researchers blinded to the experimental groups, and the scores for each rat at each time point represent the average of all observers.

#### 2.4.3 Histological analysis

Histological analysis of spinal cord tissues from the four groups was conducted using hematoxylin and eosin (H&E) staining. Tissues were fixed in 4% paraformaldehyde, dehydrated, embedded, sectioned into 5 μm paraffin slices, and stained with H&E. After cleaning, coverslipping, and drying, pathological changes in the spinal cord tissues were observed under a microscope.

#### 2.4.4 Sirius red staining

The spinal cord tissue slices were routinely deparaffinized in water, removed, stained with 0.1% Sirius Red for 1–2 min, and rinsed under running water. Subsequently, the sections were immersed in distilled water for a period, dehydrated in 75% and 95% ethanol for 5 min each, air‐dried, and sealed with neutral gum. Finally, after drying, observations were made using a conventional optical microscope.

### 2.5 Biocompatibility analysis of β‐GP/CS@ATRA

#### 2.5.1 In vivo pharmacokinetic evaluation

At different time points post‐treatment (5 min, 0.5 h, 1 h, 1.5 h, 2 h, 3 h, 4 h, 6 h, 8 h, 10 h, and 12 h), five rats from the ATRA group and β‐GP/CS@ATRA group were randomly selected. Blood samples were collected from their orbital veins. It is worth noting that, unless otherwise specified, all subsequent blood samples in the experiments were obtained from the orbital veins of rats. The blood samples were centrifuged at 3500 rpm for 15 min to obtain plasma. Then, 0.2 mL of the sample was mixed with 5 mL of ethyl acetate, followed by vortexing and centrifugation to remove plasma proteins. The samples were dried, reconstituted with the mobile phase, and injected into the High‐Performance Liquid Chromatography (HPLC) for analysis. PKSolver pharmacokinetic software was utilized to calculate the pharmacokinetic parameters.

#### 2.5.2 In vivo drug release

After treatment, blood samples were collected from randomly selected rats in the ATRA group and β‐GP/CS@ATRA group at 1, 2, 4, 6, 8, 12, 24, 36, and 48 h. Following sample processing, the concentration of ATRA was determined using high‐performance liquid chromatography (HPLC, Agilent 1260, USA) under both 4 and 37°C conditions. Drug release curves were generated based on the measured concentrations.

#### 2.5.3 Hemolysis analysis

Blood compatibility of β‐GP/CS@ATRA was assessed using an in vitro hemolysis assay.^37^ Four rats from the Control group were randomly selected, and their plasma samples were collected. The collected plasma samples from the Control group were placed in tubes containing heparin, centrifuged at 2000 rpm for 15 min, and washed and purified with 0.150 Mm NaCl. The red blood cell suspension was diluted tenfold with PBS buffer (pH 7.4). A 0.5 mL aliquot of the diluted red blood cells was mixed with 0.5 mL of β‐GP/CS@ATRA at different concentrations (10, 20, 50, 100, 200 μg/mL). Deionized water served as the positive control, and PBS solution as the negative control. Finally, all test samples were incubated at 37°C for 10 min, centrifuged at 10000 rpm, and the supernatant was collected to measure absorbance at 570 nm. The hemolysis rate was calculated using the formula: Hemolysis (%) = (A_sample – A_negative)/(A_positive – A_negative) × 100%.

#### 2.5.4 Cell culture

Human umbilical vein endothelial cells (HUVECs) were obtained from iCell Bioscience Inc. (Shanghai, China). The cells were cultured in a medium containing 5% FBS, 100 IU/mL penicillin, and 100 μg/mL streptomycin, and maintained in a 37°C, 5% CO_2_ incubator.

#### 2.5.5 Cell viability analysis

To assess the potential toxicity of β‐GP/CS@ATRA, human umbilical vein endothelial cells (HUVECs), one of the most abundant cell types in the wound tissue responsible for wound healing, were subjected to the MTT assay.^38^, ^39^ Briefly, HUVECs were seeded in a 96‐well plate (1 × 10^4^ cells/well) and incubated for 1 day. HUVECs were then treated with different concentrations of ATRA and β‐GP/CS@ATRA (0, 0.1, 1, 5, 10, 50, 100, and 150 μg/mL) for a specified period. After removing the culture medium, 20 μL of MTT solution (5 mg/mL) was added to each well, and the cells were incubated for 4 h. The supernatant was then discarded, and 150 μL of dimethyl sulfoxide was added to each well. The optical density at 570 nm was measured using a microplate reader, and cell viability was calculated using the formula: Cell viability (%) = (optical density of reacted cells)/(optical density of the control) × 100%.

#### 2.5.6 Serum biochemical analysis measurement

Twelve healthy SD rats were randomly divided into the Control group and the β‐GP/CS@ATRA group, with 6 rats in each group. The β‐GP/CS@ATRA group received β‐GP/CS@ATRA treatment, while the Control group did not receive any treatment. After 5, 10, and 15 days of treatment in the β‐GP/CS@ATRA group, we selected six rats at each time point and collected their blood samples. Simultaneously, six rats were randomly chosen from the Control group, and their blood samples were also collected. Following the previously described method,^40^ the levels of aspartate aminotransferase (AST), alanine aminotransferase (ALT), blood urea nitrogen (BUN), and creatinine (CRE) in the serum were measured using an automated chemical analyzer.

#### 2.5.7 Histological analysis

After euthanizing rats in the Control group and the β‐GP/CS@ATRA group, heart, lung, liver, spleen, and kidney tissue samples were collected. The tissue sections of the heart, lung, liver, spleen, and kidney from rats in the Control and β‐GP/CS@ATRA groups were assessed for histopathological changes using H&E staining.

### 2.6 Statistical analysis

All experiments were conducted with a minimum of three replicates. Data are presented as mean ± standard deviation. Statistical analyses were performed using GraphPad Prism 9 software (GraphPad Inc., San Diego, CA, USA). Student's t‐test was employed for comparisons between two groups, while one‐way analysis of variance (ANOVA) was used for comparisons among multiple groups. *P*‐value<0.05 was considered statistically significant.

## 3 RESULTS

### 3.1 Pin1, PI3K, AKT, and CDK2 were all upregulated in the spinal cord tissues of SCI rats, and there was a confirmed interaction between Pin1 and the PI3K/AKT/CDK2 pathway

**FIGURE 1:**
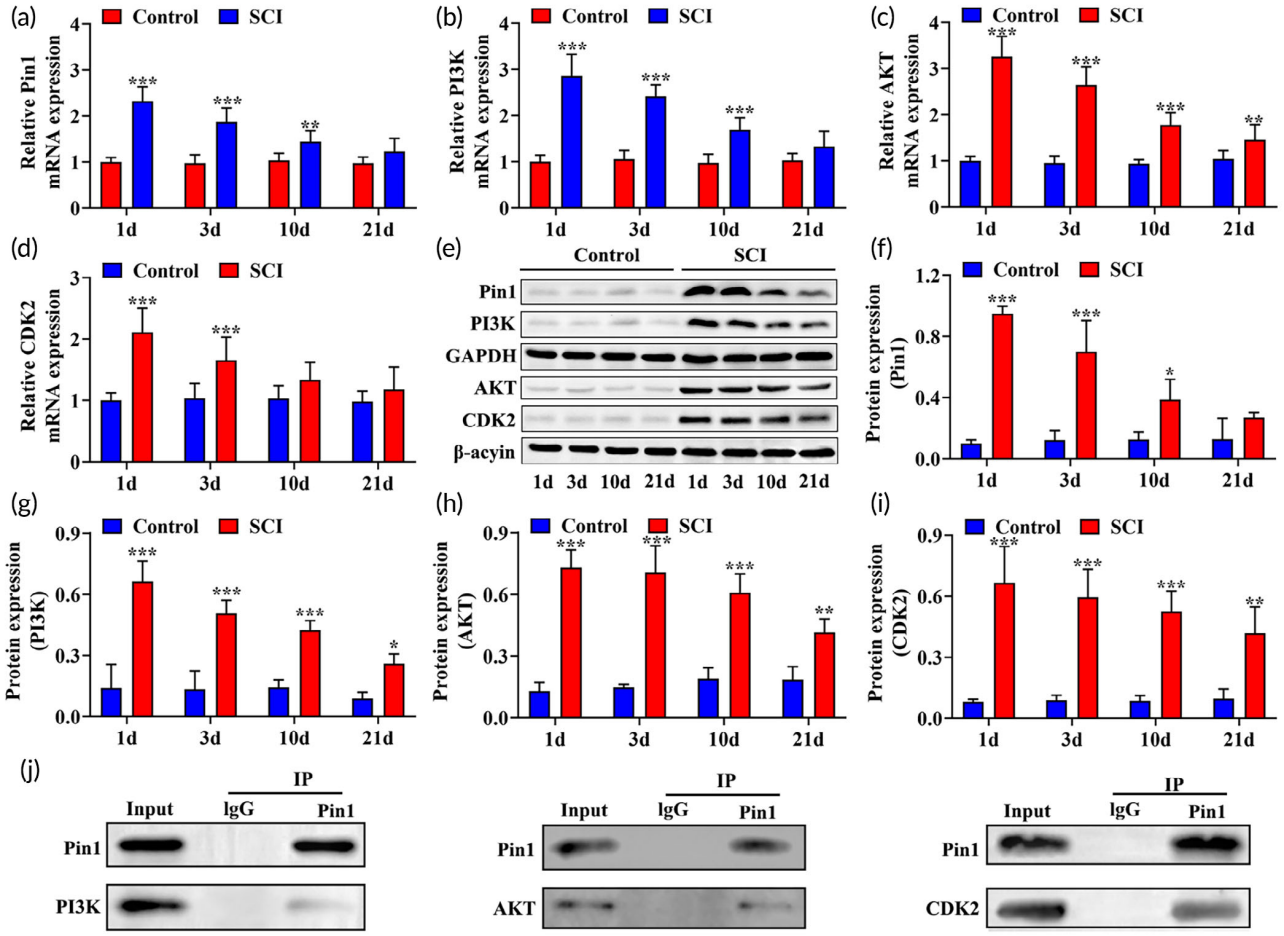
Expression and interrelationship of Pin1, PI3K, AKT, and CDK2 in spinal cord tissue after SCI. (a–d), qRT‐PCR analysis of Pin1, PI3K, AKT, and CDK2 expression in spinal cord tissue of SCI rats (*n* = 3). (e–i), Western blot assay of Pin1, PI3K, AKT, and CDK2 expression in spinal cord tissue of SCI rats (*n* = 3). (j) Co‐IP analysis detecting the interaction between Pin1 and PI3K, AKT, and CDK2 in spinal cord tissue of SCI rats (*n* = 3). *p < 0.05 versus Control group at the same time point, **p < 0.01 versus Control group at the same time point, ***p < 0.001 versus Control group at the same time point.

Pin1 is known to regulate various neuronal processes and plays a crucial role in the development of SCI.^16^ Glial scar formation is a significant impediment to neural regeneration following SCI, with astrocytes being the primary cellular component of the glial scar. After SCI, astrocytes exhibit increased proliferative capacity, thereby promoting the formation of glial scars. This process is primarily regulated by brain‐derived neurotrophic factor (BDNF) and modulated by the PI3K/AKT signaling pathway.^41^ Activation of the PI3K/AKT pathway can enhance the proliferation and migration of astrocytes, expediting the formation of glial scars.^42^ Additionally, growth‐associated protein 43 (GAP‐43) serves as a neuron‐specific calcium‐binding protein and actin‐binding protein, commonly used as a critical marker for neuronal development, axonal regeneration, and synaptic remodeling after neural injury. GAP‐43 directly participates in the regulation of intrinsic axonal growth capacity.^43^ Studies have indicated that olomoucine (a CDK2 inhibitor) can reduce spinal cavity formation, improve functional deficits, accelerate the expression of GAP‐43 in the SCI region, and indicate the involvement of CDK2 in SCI development, with the PI3K/AKT pathway being necessary for CDK2 activation.^22^, ^44^ Consistent with existing research findings, qRT‐PCR and western blot analyses in this study revealed elevated expression levels of Pin1, PI3K, AKT, and CDK2 in the SCI group compared to the Control group at 1, 3, 10, and 21 days post‐SCI induction (Figure 1a–i). Additionally, immunofluorescence results also revealed a significant increase in the expression level of Pin1 in spinal cord tissue (Figure S1). These results suggest the involvement of Pin1 and the PI3K/AKT/CDK2 pathway in glial scar formation. Furthermore, Co‐IP results demonstrated the interaction between Pin1 and PI3K, AKT, and CDK2 (Figure 1j), indicating a mutual relationship between Pin1 and the PI3K/AKT/CDK2 pathway.

### 3.2 Pin1 promotes the proliferation of astrocytes, inhibits their apoptosis, and activates the PI3K/AKT/CDK2 pathway

**FIGURE 2:**
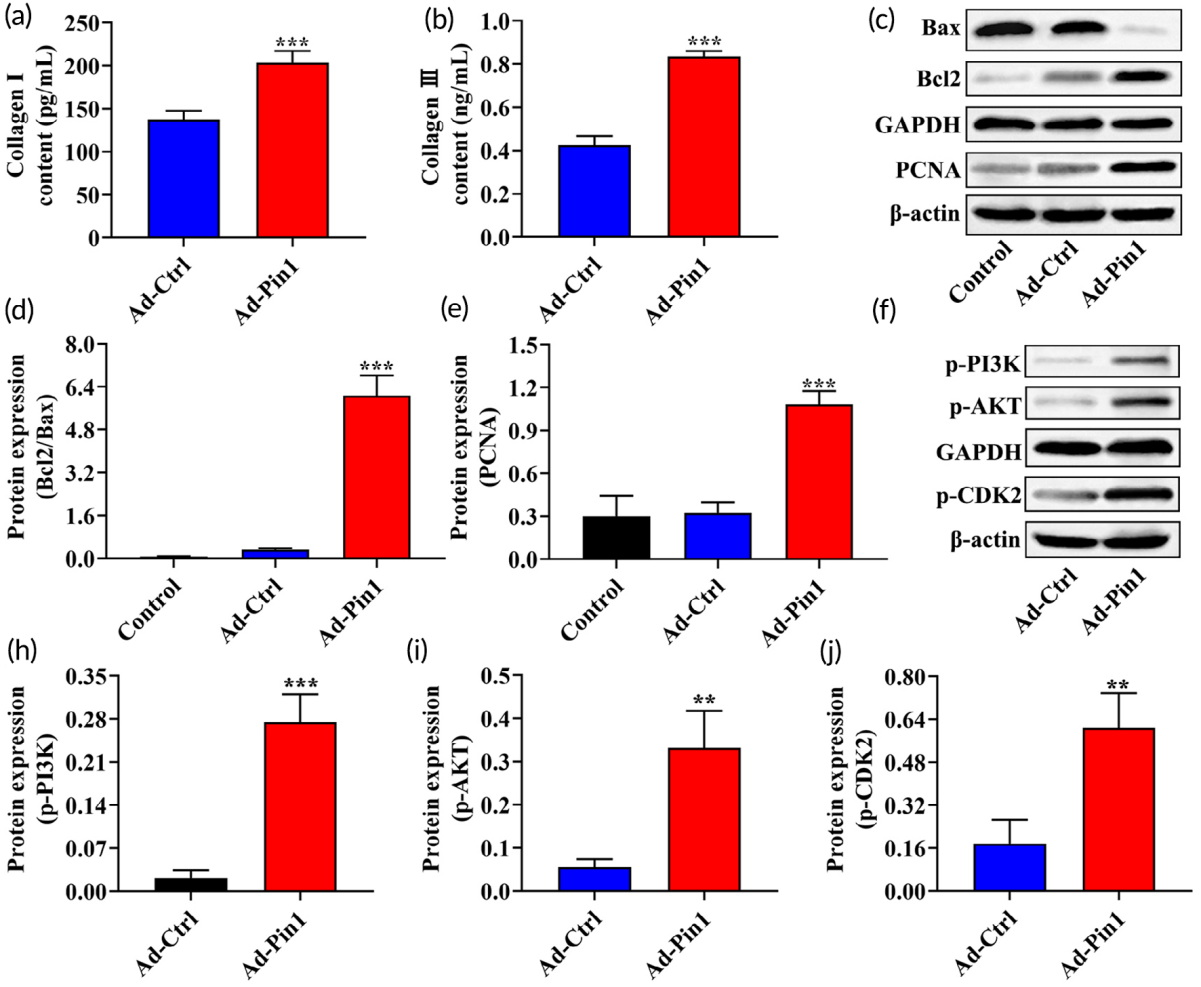
Effects of Pin1 on astrocytes and the PI3K/AKT/CDK2 pathway. (a, b) ELISA assay to measure the expression levels of collagen I and collagen III in astrocytes. (c–e) Western blot assay to evaluate the expression levels of apoptosis‐related proteins (Bax, Bcl2) and proliferation‐related protein (PCNA) in astrocytes. (f–j) Western blot assay to examine the expression levels of p‐PI3K, p‐AKT, and p‐CDK2 proteins in astrocytes. **p < 0.01 versus Ad‐Ctrl, ***p < 0.001 versus Ad‐Ctrl.

Astrocytes can stabilize glial scars by producing extracellular matrix (ECM) molecules.^45^ The ECM in the spinal cord consists of various components, including collagens, proteoglycans, and glycoproteins.^46^ Studies have shown that collagen I is highly expressed in the spinal cord during the scar formation stage and induces astrocytic scar formation through the integrin‐N‐cadherin pathway. Thus, collagen I is directly involved in the process of glial scar formation.^47^ Our previous findings demonstrated that Pin1 expression is significantly elevated in SCI and interacts with the PI3K/AKT/CDK2 pathway. To further investigate the role of Pin1 in glial scars formation, we isolated primary astrocytes from the spinal cord tissue of SD rats and detected the localization of Pin1 in astrocytes using immunofluorescence staining. Additionally, we used adenovirus infection to overexpress Pin1 in astrocytes (Figure S2). The effects of Pin1 overexpression on collagen expression (collagen I and III), astrocyte proliferation, and apoptosis were assessed by ELISA and Western blot analysis. Immunofluorescence staining revealed that Pin1 is primarily localized in the nucleus and cytoplasm of astrocytes (Figure S3). ELISA results showed that, compared to the Ad‐Ctrl group, the expression levels of collagen I and collagen III were significantly increased in the Ad‐Pin1 group (*p* < 0.001, Figure 2a,b), suggesting that Pin1 promotes glial scar formation by enhancing the expression of collagen I and collagen III. Western blot analysis demonstrated that overexpression of Pin1 significantly increased the Bcl2/Bax ratio (apoptosis‐related proteins) and the expression level of PCNA (proliferation‐related protein) (*p* < 0.001, Figure 2c–e), indicating that Pin1 promotes astrocyte proliferation and inhibits apoptosis. Furthermore, to explore the relationship between Pin1 and the PI3K/AKT/CDK2 pathway, we performed additional Western blot analysis. The results showed that overexpression of Pin1 significantly upregulated the expression levels of p‐PI3K, p‐AKT, and p‐CDK2 (Figure 2f–j), suggesting that Pin1 activates the PI3K/AKT/CDK2 pathway. These findings indicate that Pin1 promotes astrocyte proliferation, inhibits apoptosis, and activates the PI3K/AKT/CDK2 pathway, thereby contributing to the development of glial scars.

### 3.3 Preparation and characterization of β‐GP/CS@ATRA

**FIGURE 3:**
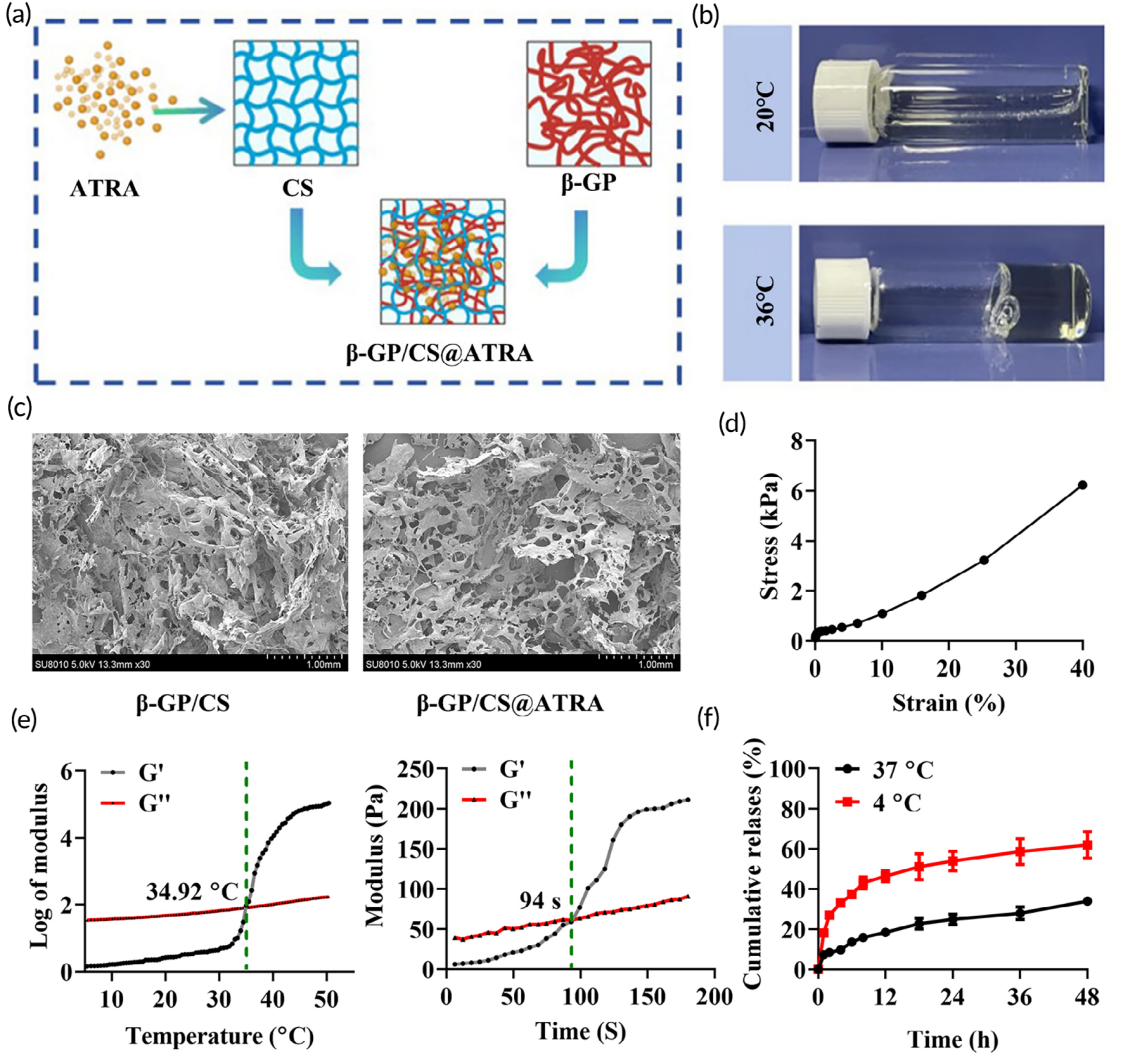
Preparation and Characterization of β‐GP/CS and β‐GP/CS@ATRA. (a) Schematic illustration of the preparation process for β‐GP/CS and β‐GP/CS@ATRA. (b) Thermal response images of the hydrogels. (c) SEM images depicting the morphology of β‐GP/CS and β‐GP/CS@ATRA hydrogels. (d) Stress–strain curve of β‐GP/CS hydrogel. (e) Modulus changes at different temperatures and times for β‐GP/CS hydrogel. (f) In vitro drug release curve for β‐GP/CS@ATRA hydrogel. G', storage modulus; G", loss modulus.

Due to the enhanced hydrogen bonding, electrostatic forces, and hydrophobic interactions between β‐GP and CS,^48^ we successfully prepared β‐GP/CS and β‐GP/CS@ATRA hydrogels, as illustrated in Figure 3a. At 20°C, the β‐GP/CS solution exhibited fluidity and did not gel. However, when the temperature increased to 36°C, the β‐GP/CS solution underwent a sol–gel transition, forming a typical solid‐state hydrogel (Figure 3b), consistent with the thermosensitive properties of hydrogels.^49^ The morphology of the β‐GP/CS and β‐GP/CS@ATRA hydrogels was investigated by SEM. SEM results revealed that both β‐GP/CS hydrogel and β‐GP/CS@ATRA hydrogel exhibited irregular porous structures with pore sizes of approximately 100–250 μm (Figure 3c). This indicates that loading ATRA does not affect the morphology of β‐GP/CS, and the porous structure is advantageous for the release of ATRA.^48^ To investigate the biomechanical properties of the gel, we plotted the compression stress–strain curve of the β‐GP/CS hydrogel and measured the modulus changes at different times and temperatures within the elastic deformation region. The results indicated that deformation less than 10% falls within this elastic region (Figure 3d). Therefore, we selected a 10% strain to evaluate the modulus changes at various times and temperatures. The results revealed that the phase transition temperature of the β‐GP/CS hydrogel is 34.92°C, with a phase transition time of 94 s (Figure 3e). Additionally, in vitro drug release results of the β‐GP/CS@ATRA hydrogel showed that under 37°C conditions, compared to 4°C, the drug release from β‐GP/CS@ATRA hydrogel was smoother and lasted longer (Figure 3f), indicating that the sustained‐release capability of β‐GP/CS@ATRA contributes to the long‐term efficacy of the drug.

### 3.4 Inhibition of glial scar formation and activation of the PI3K/AKT/CDK2 pathway by β‐GP/CS@ATRA in vivo

**FIGURE 4:**
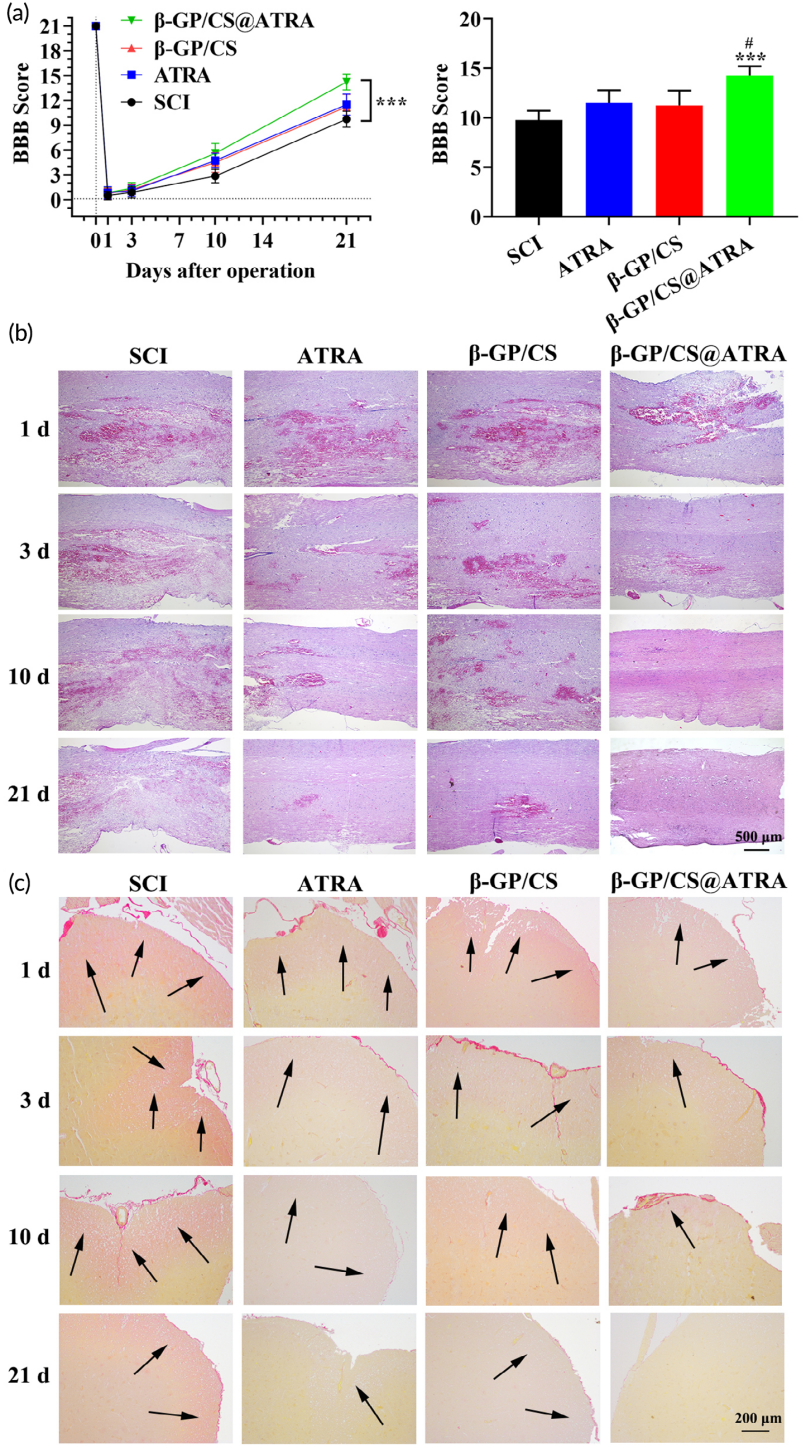
Inhibition of glial scar formation by β‐GP/CS@ATRA in vivo. (a) BBB scores of rats from four groups at 21 days post‐injury (*n* = 3). (b) H&E staining of spinal cord tissue sections from four groups of rats (×200) (*n* = 3), scale bar = 500 μm. (c) Sirius Red staining of spinal cord tissue sections from four groups of rats, with black arrows indicating areas of dense collagen deposition (×200) (*n* = 3), scale bar = 200 μm. ***p < 0.001 versus SCI, ^#^ *p* < 0.05 versus ATRA.

**FIGURE 5:**
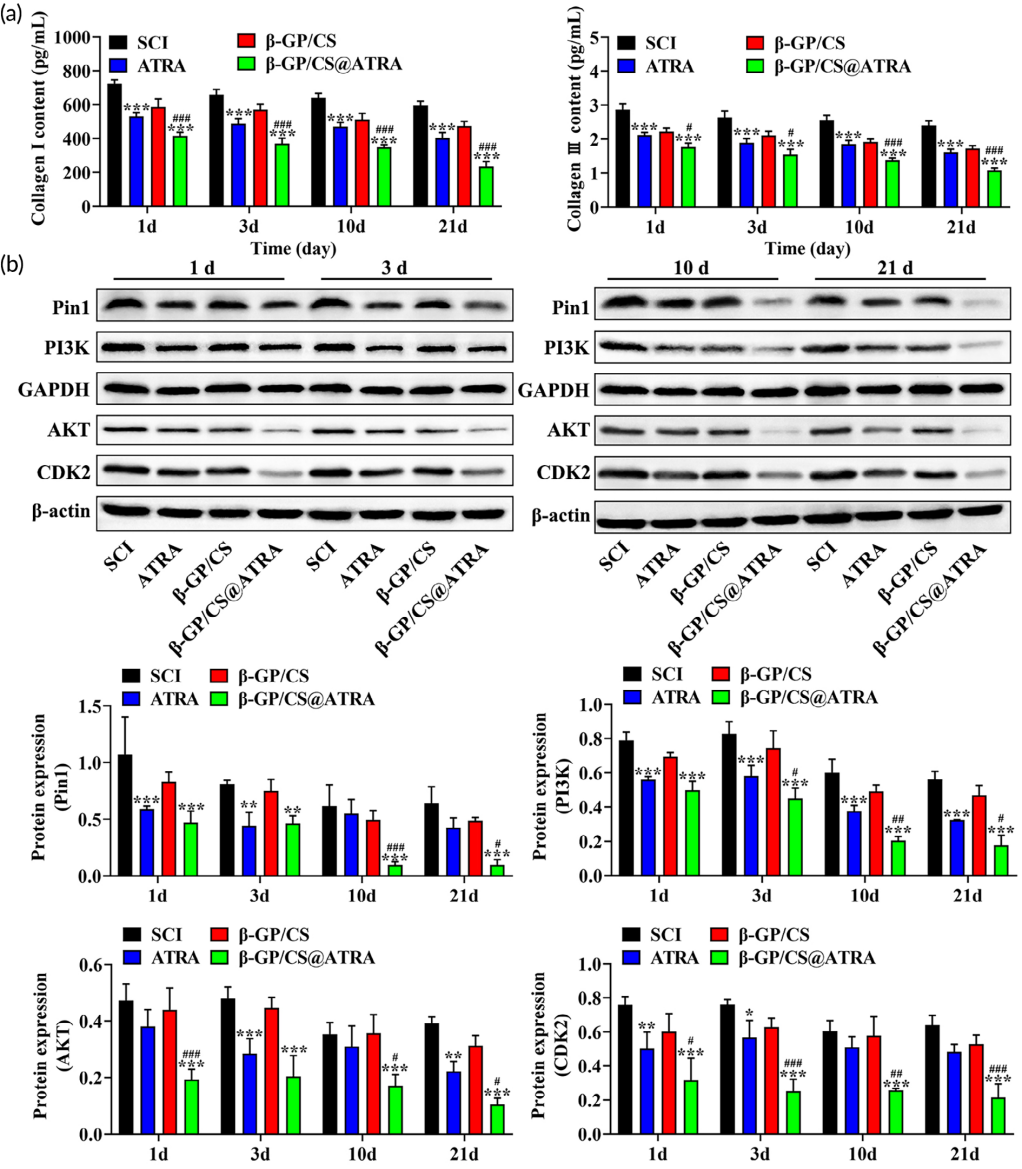
Inhibition of Pin1 expression and suppression of PI3K/AKT/CDK2 pathway activation by β‐GP/CS@ATRA in vivo. (a) ELISA analysis of the expression levels of collagen I and collagen III in blood samples (*n* = 3). (b) Western blot assay to detect the expression levels of Pin1, PI3K, AKT, and CDK2 in blood samples (*n* = 3). *p < 0.05 versus SCI, **p < 0.01 versus SCI, ***p < 0.001 versus SCI, ^#^ *p* < 0.05 versus ATRA, ^##^ *p* < 0.01 versus ATRA, ^###^ *p* < 0.001 versus ATRA.

We have successfully obtained a thermosensitive β‐GP/CS@ATRA, and next, we investigated the in vivo therapeutic effect of β‐GP/CS@ATRA on glial scar formation. To evaluate the efficacy of β‐GP/CS@ATRA on glial scar, we transplanted β‐GP/CS@ATRA hydrogel into the SCI model of SD rats. First, within 3 weeks post‐injury, the locomotor recovery of rats was assessed using the BBB scale. As shown in Figure 4a, after 10 days post‐surgery, the motor function gradually improved in all groups, with BBB scores increasing slightly over time in the ATRA and β‐GP/CS@ATRA groups compared to the SCI and β‐GP/CS groups, but without statistical significance. After 21 days post‐surgery, the SCI group showed slow improvement in BBB scores, while the ATRA and β‐GP/CS groups had slightly higher scores than the SCI group. Moreover, the BBB scores of the β‐GP/CS@ATRA group were significantly higher than the SCI group (*p* < 0.001, Figure 4a), indicating that the implantation of ATRA‐loaded β‐GP/CS hydrogel had the optimal effect on the locomotor recovery of SCI rats. Additionally, to observe the effect of β‐GP/CS@ATRA hydrogel on spinal cord tissue, we performed H&E staining and Sirius Red staining. H&E and Sirius Red staining results (Figure 4b,c) revealed that both the SCI group and β‐GP/CS group exhibited disordered spinal cord tissue arrangement, spinal cord cavitation, glial scar formation, and collagen deposition. After administration of ATRA, these phenomena were alleviated, with the β‐GP/CS@ATRA group showing better results compared to the ATRA group. This suggests that, compared to ATRA alone, β‐GP/CS@ATRA can better protect spinal tissue and reduce glial scar formation. Furthermore, previous studies have shown that Pin1 can upregulate the expression of collagen I, collagen III, PI3K, AKT, and CDK2. To investigate the impact of ATRA on these proteins and Pin1 expression levels, we conducted ELISA and western blot assays. ELISA analysis revealed that, compared to the SCI group, the expression of collagen I and collagen III in the ATRA and β‐GP/CS@ATRA groups significantly decreased (*p* < 0.001). The downregulation of collagen I and collagen III expression in the β‐GP/CS@ATRA group was more pronounced than that in the ATRA group alone (Figure 5a). Western blot assay results showed that after ATRA treatment, the expression levels of Pin1, PI3K, AKT, and CDK2 decreased, with the β‐GP/CS@ATRA group showing the most significant effect (Figure 5b). In summary, these results suggest that ATRA can hinder the development of glial scars and the activation of the PI3K/AKT/CDK2 pathway in vivo by inhibiting Pin1. Additionally, the β‐GP/CS@ATRA hydrogel exhibits a more significant effect compared to ATRA alone.

### 3.5 The β‐GP/CS@ATRA exhibits excellent biocompatibility

**FIGURE 6:**
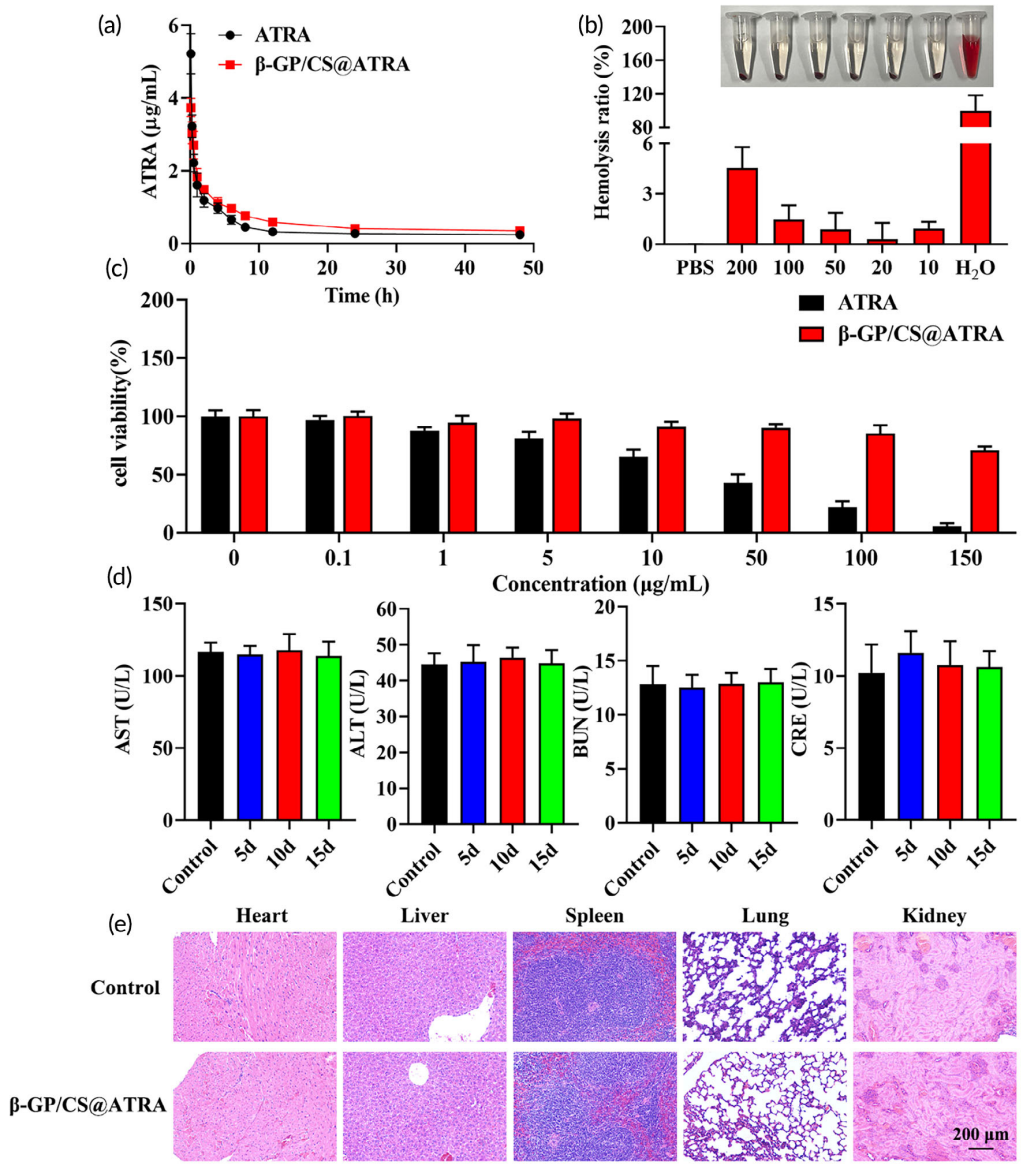
Excellent biocompatibility of β‐GP/CS@ATRA. (a) In vivo drug release curve (*n* = 3). (b) Hemolysis rate of rat blood cells treated with different concentrations of β‐GP/CS@ATRA (*n* = 4). (c) Cytotoxicity of HUVECs cells treated with different concentrations of β‐GP/CS@ATRA (*n* = 5). (d) Serum levels of AST, ALT, BUN, and CRE measured using an automatic biochemical analyzer (*n* = 6). (e) H&E staining of heart, liver, spleen, lung, and kidney tissues from two groups of rats after 21 days of administration (×200) (*n* = 3), scale bar = 200 μm. ALT, alanine aminotransferase; AST, aspartate aminotransferase; BUN, blood urea nitrogen; CRE, creatinine.

Excellent biocompatibility is a crucial prerequisite for the biomedical application of biomaterials.^50^ Therefore, we conducted a comprehensive investigation into the biocompatibility of β‐GP/CS@ATRA. Results from in vivo pharmacokinetics and drug release studies revealed that, compared to standalone ATRA, β‐GP/CS@ATRA exhibits a longer half‐life in vivo, favoring prolonged therapeutic effects of ATRA (Table 2, Figure 6a). Hemolysis assay results demonstrated that even at a concentration of 200 μg/mL, the hemolysis rate of β‐GP/CS@ATRA was below 5% (Figure 6b), indicating non‐hemolytic properties and its potential as a blood‐compatible drug delivery system. Cytotoxicity results (Figure 6c) showed that, with increasing drug dosage, the cell viability of the ATRA group gradually decreased, while the cell viability of the β‐GP/CS@ATRA group, although slightly reduced, remained above 60%, indicating the absence of cytotoxicity. Blood‐related analysis results (Figure 6d) indicated that levels of alanine transaminase (ALT), aspartate transaminase (AST), blood urea nitrogen (BUN), and creatinine (CRE) in the β‐GP/CS@ATRA group remained within normal ranges and were comparable to those in the Control group, suggesting that loading ATRA onto β‐GP/CS does not impair liver or kidney function. Additionally, HE staining results (Figure 6e) revealed no apparent tissue damage or morphological abnormalities in the heart, liver, spleen, lung, and kidney tissues of the β‐GP/CS@ATRA group, demonstrating the safety of in vivo application of β‐GP/CS@ATRA. In summary, these findings indicate that β‐GP/CS@ATRA possesses excellent biocompatibility, highlighting its potential clinical utility.

**TABLE 2:** Pharmacokinetic parameters of β‐GP/CS@ATRA and ATRA in vivo.

Parameters	Unit	ATRA	β‐GP/CS@ATRA
*t*_1/2β_	h	5.03 ± 2.07	17.77 ± 4.55
AUC_0–t_	μg h mL^−1^	13.05 ± 2.02	25.94 ± 3.64
AUC_0–∞_	μg h mL^−1^	13.11 ± 2.11	26.73 ± 1.52
MRT_0–∞_	h	6.63 ± 2.77	20.48 ± 3.00
Vd	L kg^−1^	0.87 ± 0.13	1.22 ± 0.14
CL	L h^−1^ kg^−1^	0.59 ± 0.08	0.31 ± 0.06

## 4 CONCLUSION

In summary, our study elucidated that Pin1 plays a pivotal role in the occurrence and development of glial scar formation following SCI by promoting the proliferation and inhibiting the apoptosis of astrocytes, along with the activation of the PI3K/AKT/CDK2 pathway. Additionally, we successfully prepared β‐GP/CS@ATRA, an ATRA‐loaded temperature‐sensitive β‐GP/CS hydrogel, for the first time. In vivo investigations demonstrated that β‐GP/CS@ATRA impedes the development of glial scars and the activation of the PI3K/AKT/CDK2 pathway by suppressing Pin1 expression, exhibiting favorable biocompatibility. These findings suggest that Pin1 may serve as a potential therapeutic target for mitigating glial scar formation post‐SCI. Furthermore, the β‐GP/CS@ATRA hydrogel exhibits promising applications, representing a prospective targeted treatment for glial scar formation after SCI.

## AUTHOR CONTRIBUTIONS

**Rongmou Zhang:** Investigation; methodology; writing – original draft. **Ting Tang:** Investigation; methodology; writing – original draft. **Huafeng Zhuang:** Formal analysis; investigation; resources. **Peiwen Wang:** Investigation; resources. **Haiming Yu:** Formal analysis; validation. **Hao Xu:** Validation; visualization. **Xuedong Yao:** Conceptualization; validation; writing – review and editing.

## FUNDING INFORMATION

The project was funded by the Fujian Provincial Natural Science Foundation Project (Grant no. 2020J01230 and 2020J01235) and Quanzhou City Science & Technology Program of China (Grant no. 2023C003YR).

## CONFLICT OF INTEREST STATEMENT

The authors declare no conflicts of interest.

## Supporting information


**Figure S1.** Immunofluorescence detection of Pin1 expression in spinal cord tissue of rats with spinal cord injury (SCI). The images were scanned at 40× magnification.
**Figure S2.** Effect of adenoviral infection on primary astrocytes. (A) qRT‐PCR analysis. (B) Western blot analysis. All experiments were performed three times. ***p < 0.001 versus Ad‐Ctrl.
**Figure S3.** Immunofluorescence detection of Pin expression in astrocytes. The images were scanned at 400× magnification.

## Data Availability

The data that support the findings of this study are available from the corresponding author upon reasonable request.
