# Supplementary material for: Temperature‐sensitive sodium beta‐glycerophosphate/chitosan hydrogel loaded with all‐trans retinoic acid regulates Pin1 to inhibit the formation of spinal cord injury‐induced rat glial scar
Source: Bioeng Transl Med. 2024 Oct 17;10(3):e10729. doi: 10.1002/btm2.10729 (PMC12079469; doi:10.1002/btm2.10729)
Supplement: Supplementary file 1 — Figure S1. Immunofluorescence detection of Pin1 expression in spinal cord tissue of rats with spinal cord injury (SCI). The images were scanned at 40× magnification. Figure S2. Effect of adenoviral infection on primary astrocytes. (A) qRT‐PCR analysis. (B) Western blot analysis. All experiments were performed three times. ***p < 0.001 versus Ad‐Ctrl. Figure S3. Immunofluorescence detection of Pin expression in astrocytes. The images were scanned at 400× magnification. [file BTM2-10-e10729-s001.docx]

**Figure S1 Immunofluorescence detection of Pin1 expression in spinal cord tissue of rats with spinal cord injury (SCI).** The images were scanned at 40×magnification.

**
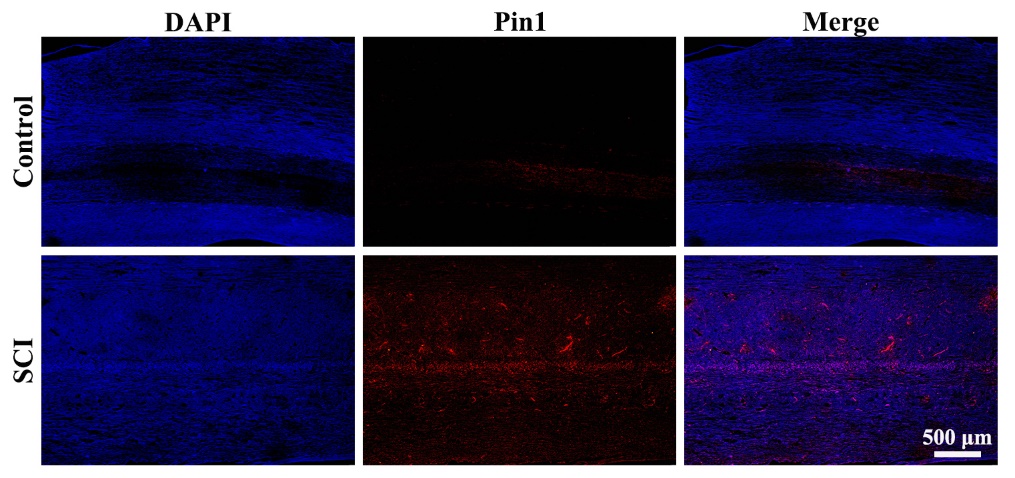
**

**Figure S2 Effect of adenoviral infection on primary astrocytes.** A, qRT-PCR analysis. B, Western blot analysis. All experiments were performed three times. ^***^*P*<0.001 vs Ad-Ctrl.


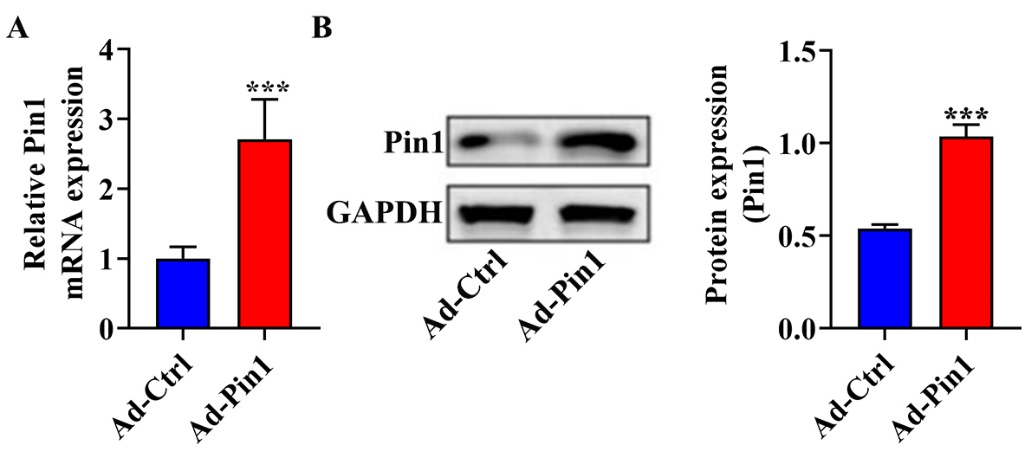


**Figure S3 Immunofluorescence detection of Pin expression in astrocytes.** The images were scanned at 400×magnification.

**
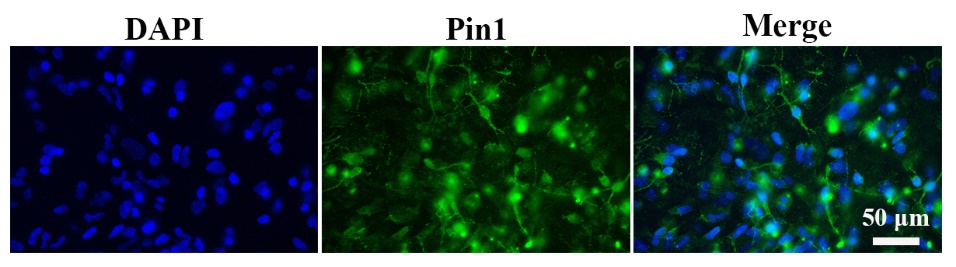
**
